# Supplementary material for: Disabling VEGF-Response of Purkinje Cells by Downregulation of KDR via miRNA-204-5p
Source: Int J Mol Sci. 2021 Feb 22;22(4):2173. doi: 10.3390/ijms22042173 (PMC7926311; doi:10.3390/ijms22042173)
Supplement: Supplementary file 1 [file ijms-22-02173-s001.pdf]

Supplementary Materials

Disabling VEGF-Response of Purkinje Cells by Downregulation of *KDR* via miRNA-204-5p

Julian Gehmeyr [1], Abdelouahid Maghnouj [2], Jonas Tjaden [1], Matthias Vorgerd [3], Stephan Hahn [2], Veronika Matschke [1], Verena Theis [1] and Carsten Theiss [1,*]

[1] Department of Cytology, Institute of Anatomy, Ruhr-University Bochum, Universitaetsstr. 150, Building MA, Level 5, 44780 Bochum, NRW, Germany; julgeh@t-online.de (J.G.); Jonas.Tjaden@ruhr-uni-bochum.de (J.T.); veronika.matschke@rub.de (V.M.); Verena.theis@rub.de (V.T.)

[2] Clinical Research Centre (ZKF), Department of Molecular Gastrointestinal Oncology, Ruhr-University Bochum, Universitaetsstr. 150, 44801 Bochum, Germany; abdelouahid.maghnouj@rub.de (A.M.); stephan.hahn@rub.de (S.H.)

[3] Neuromuscular Center Ruhrgebiet, Department of Neurology, University Hospital Bergmannsheil, Ruhr-University Bochum, Buerkle-de-la-Camp-Platz 1, 44789 Bochum, Germany; matthias.vorgerd@bergmannsheil.de

* Correspondence: carsten.theiss@rub.de; Tel.: +49-234-32-24560

Contents

- **Supplementary Figure S1:** Cerebellar cortex in cryosections and organotypic slices.
- **Supplementary Figure S2:** PC morphology during development in cryosections and organotypic slices.

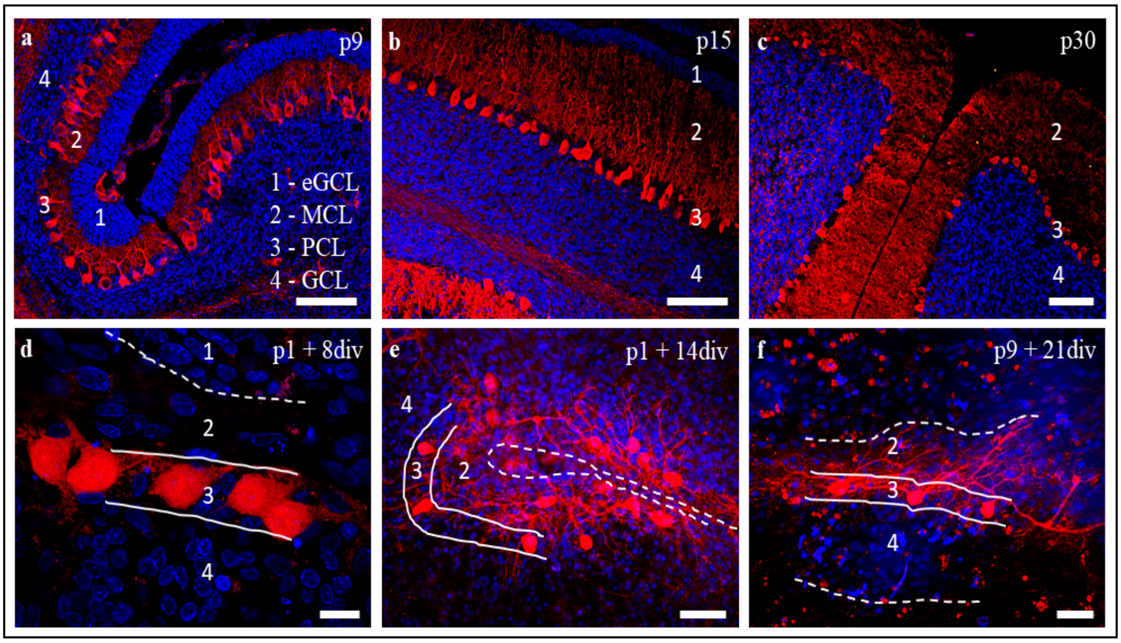

Supplementary Figure S1. (**a**–**c**) Cerebellar cryosections: p9, p15, p30; (**d**–**f**); cerebellar slice cultures: p1 + 8div, p1 + 14div, p9 + 21div. All images show calbindin positive PC (red) and cell nuclei (blue). (**a**, **b**) In p9 neonatal and p15 juvenile cryosections the four-layered cortex structure of the cerebellum is displayed by the staining, also visible at (**d**) p1 + 8div. Therein, extra granule cells migrate from the external granular cell layer (eGCL) to the granular cell layer (GCL) in the course of cerebellar development. (**c**, **f**) In mature PC, the migration has finished, and PC have formed a thick molecular cell layer (MCL) with their extended dendritic trees. The Purkinje cell layer (PCL) itself shows a characteristic line shape. (**d**–**f**) Although, in the organotypic slice cultures, the natural arrangement of cells has loosened, characteristic cerebellar structures are still clearly visible; PC develop an age-appropriate dendritic tree and keep their cell-cell contacts. Scale bars: (**a**–**c**) 100 μm; (**d**) 20μm; (**e**, **f**) 50 μm.

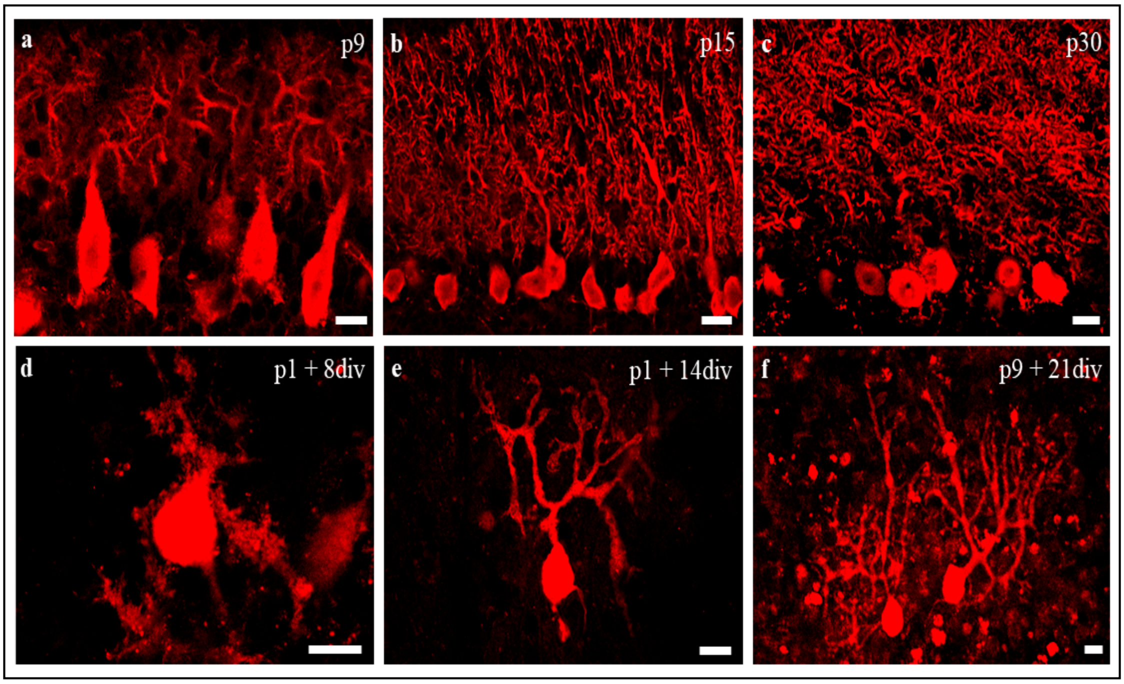

Supplementary Figure S2. All images show calbindin-positive PCs stained with red fluorescent protein. (**a**–**c**) Cerebellar cryosections: p9, p15, p30: During maturation, PC develop from (**a**) a simple bipolar shape with small early main dendrites (**b**) building secondary and single tertiary branches to (**c**) mature PC at p30 showing a characteristic heavily branched dendritic tree with numerous dendritic spines. (**d**–**f**) Cerebellar slice cultures: p1 + 8 div; p1 + 14 div; p9 + 21 div. In organotypic slice cultures, the dendritic tree is expanded quite similar during development. Scale bars: 20 μm.
